# Contribution of kelp dashi liquid to sustainable maintenance of taste sensation and promotion of healthy eating in older adults throughout the umami-taste salivary reflex

**DOI:** 10.3389/fnut.2024.1406633

**Published:** 2024-08-27

**Authors:** Shizuko Satoh-Kuriwada, Satoshi Gotoh, Noriaki Shoji, Hisayuki Uneyama, Michio Komai

**Affiliations:** ^1^Division of Comprehensive Dentistry, Tohoku University Hospital, Sendai, Japan; ^2^Haneishi Dental Clinic, Utsunomiya, Japan; ^3^Shoji Dental Clinic, Sendai, Japan; ^4^Corporate Division, Department of Global Communication, Ajinomoto Co., Inc., Tokyo, Japan; ^5^Laboratory of Nutrition, Graduate School of Agricultural Science, Tohoku University, Sendai, Japan

**Keywords:** kelp dashi liquid, umami-taste loss, recognition thresholds of five basic tastes, older adults, umami-taste salivary reflex, subjective eating and swallowing difficulties, minor salivary gland flow rate

## Abstract

**Introduction:**

Taste decline, including taste loss in older adults, leads to malnutrition and frailty. In a super-aging society, improving taste decline and maintaining taste sensation are crucial for the wellbeing of older adults. Hyposalivation frequently affects older individuals and is the leading cause of taste decline in older adults. Treating taste decline, including taste loss, in older adults presents challenges due to the limited sustainable methods for increasing saliva production, except for drug therapy, which may lead to adverse effects. Umami-taste stimulation results in a prolonged increase in both the whole salivary flow rate (WF), more than 90% of which is secreted from the major salivary glands, and the minor salivary gland flow rate (MF) in healthy volunteers through the umami-taste salivary reflex. We hypothesized that umami-rich kelp dashi liquid (KDL), commonly used in Japanese cuisine, may alleviate taste decline and sustain normal taste sensation in older adults with hyposalivation. This study investigated whether KDL stimulation could improve taste decline.

**Materials and methods:**

A non-randomized controlled trial was conducted at the dental department of a university hospital, involving those who presented with dry mouth between May 2017 and December 2021. Before and after repeated KDL stimulation, characteristics like changes in WF and MF, the recognition thresholds (RTs) for five basic tastes, and subjective eating and swallowing difficulties were assessed. Statistical comparisons were performed between the values measured before and after KDL stimulation.

**Result:**

A total of 35 older patients were included. Patients with reduced MF and with or without reduced WF exhibited umami-taste loss. Repeated stimulation with KDL increased MF and WF and improved taste loss, including umami, decreased RTs, and normalized each taste. Furthermore, subjective taste impairment, subjective eating and swallowing difficulties, and burning sensations in the oral mucosa were alleviated.

**Conclusion:**

These findings indicate that KDL stimulation improved umami-taste loss and normalized each taste sensation, further alleviating eating difficulties via the umami-taste salivary reflex. Importantly, umami-taste loss was also observed in patients with normal WF but decreased MF, who are typically not diagnosed with hyposalivation. Therefore, KDL has the potential to sustain taste sensations and promote healthy eating habits in older individuals.

## 1 Introduction

Taste decline, including taste loss in older adults, can result in inadequate nutritional intake (1) and diminished appetite, ultimately leading to frailty (2, 3). Frailty represents a transitional state between a healthy and dependent state, and with suitable interventions, it can be reversed to restore health. Therefore, addressing taste loss through treatment to restore normal taste sensation could mitigate malnutrition, help older adults regain wellbeing, and reverse frailty. In a super-aging society, addressing taste loss and preserving normal taste sensations have emerged as critical concerns for the wellbeing of older individuals (4). In contrast, hyposalivation is a prevalent symptom among older adults and remains a leading cause of taste loss in this demographic (5). It has been reported that the whole salivary flow rate (WF) is decreased in older individuals with hypogeusia (6). This condition often results from causes such as the side effects of prescription drugs and age-related degeneration of the salivary glands, among others (7–9). Hyposalivation is also associated with malnutrition and frailty (6, 10, 11). Taste decline and taste loss are strongly related to hyposalivation for the following reasons: (1) impaired arrival of tastants to the taste pores and microvilli of taste buds due to decreased saliva, and (2) impairment of taste receptors due to a decrease in organic components contained in saliva (mucous glycoprotein, secretory immunoglobulin A [IgA], growth factor, etc.) (10). Consequently, frailty reported to be associated with hyposalivation may be related to taste decline and taste loss. Increasing saliva levels may help improve taste decline and loss and maintain normal taste sensation in older adults, given that hyposalivation is a common symptom in this demographic.

Saliva is secreted from the major and minor salivary glands. Reduced WF has been the primary focus when treating dry mouth, as the majority of whole saliva, approximately 90% or more of whole saliva, originates from the major salivary glands. However, in recent times, there has been a growing recognition of the presence of dry mouth with normal WF (12–14), which is traditionally not diagnosed as hyposalivation. This has shifted attention toward the MF. Although the minor salivary glands contribute to approximately 8% of whole saliva (15), they are widely distributed throughout the oral mucosa (16). Saliva from these minor salivary glands is rich in mucin (17, 18) and secretory IgA (19). Importantly, it is continuously produced both when stimulated and during rest periods (15).

However, to date, the relationship between taste decline, including taste loss and MF has not been elucidated.

Treatment of taste decline, except for drug therapy, in older individuals is difficult due to the lack of a sustainable increase in saliva production; however, drug therapy may cause side effects. Current drug therapies for dry mouth primarily stimulate the parasympathetic nerve to enhance saliva secretion but can lead to side effects such as sweating, nausea, headaches, and vomiting (20). Older adults are more likely to experience these side effects. As a solution to this problem, treatments involving natural products such as plant extracts, including ginger (21, 22), and the treatment method involving the use of the gustatory-saliva reflex to increase the amount of saliva secretion (23–25) have been explored. Among the five basic taste stimuli, umami stimulation elevates both WF and MF to a greater extent than sweet, salty, or bitter stimuli and to a comparable extent to sour stimuli. In particular, the effects of the umami taste persist longer than those of the other four basic tastes in healthy volunteers (24, 25). Umami is effective for increasing WF and MF. Based on these results, we considered that a therapy for taste decline, including taste loss, should be developed using umami. Kelp dashi liquid (KDL), a staple of Japanese cuisine, is rich in umami due to its high glutamate acid content while also containing minimal amounts of other nutrients (26). We hypothesized that the umami-rich KDL could aid in improving taste decline, including taste loss and sustaining normal taste sensation in older adults with hyposalivation, which is a prevalent condition in this demographic.

This study aimed to investigate whether repeated stimulation with KDL could improve taste sensation by increasing MF and/or WF in older patients with dry mouth. Additionally, the research aimed to determine whether KDL stimulation could serve as a sustainable method to maintain healthy taste sensations.

## 2 Materials and methods

### 2.1 Study design and patients

To identify strategies for addressing taste decline, including taste loss in older adults, we studied the intricate relationships between hyposalivation (decreased saliva), the most common cause of taste disorders in this demographic, and taste decline. Moreover, we investigated the effectiveness of KDL stimulation as a potential method for improving taste decline. A non-randomized controlled trial was conducted, which included 54 patients with dry mouth who visited the Dentistry Division of Tohoku University Hospital with complaints of dry mouth sensation between May 2017 and December 2021. The study included patients who were ≥20 years old and had decreased minor salivary secretion. The exclusion criteria were: (i) inability to answer questions and provide written consent due to cognitive decline; (ii) oral cancer, or history of radiotherapy in the head and neck, or chemotherapy; (iii) severe ulcerate, lichenoid, or herpetiform oral mucosal disease; (iv) allergies or hypersensitivity to kelp; and (v) patients with iodine contraindication diseases, such as thyroid disease, pulmonary tuberculosis, and Juhring's dermatitis herpetiformis.

To clarify the relationship between minor salivary secretion and the efficacy of KDL in improving oral dryness, we divided patients into the following two groups: (i) the normal whole saliva secretion flow rate with low minor saliva secretion flow rate (NWS-LMS) group, in which patients only had dry mouth sensation, and (ii) the low whole saliva secretion flow rate with low minor saliva secretion flow rate (LWS-LMS) group, in which patients had hyposalivation.

The recommended sample size was calculated based on preliminary experiments using the statistical software JMP R-Pro 17. According to the results of preliminary experiments in patients, the number of patients with increased MF caused by repeated stimulation with KDL was 3 out of 5 patients in the NWS-LMS group and 4 out of 7 patients in the LWS-LMS group. Therefore, by setting the significance level to 0.05, the power to 0.8, and the hypothetical difference value to 0.2, the sample size comprised eight patients in the NWS-LMS group and 13 patients in the LWS-LMS group.

This study was approved by the Ethics Committee of the Tohoku University Graduate School of Dentistry (Ethics No. 2016-3-027) and was conducted following the Declaration of Helsinki and the Ethical Guidelines for Medical and Health Research Involving Human Subjects.

### 2.2 Procedure

**Figure 1:**
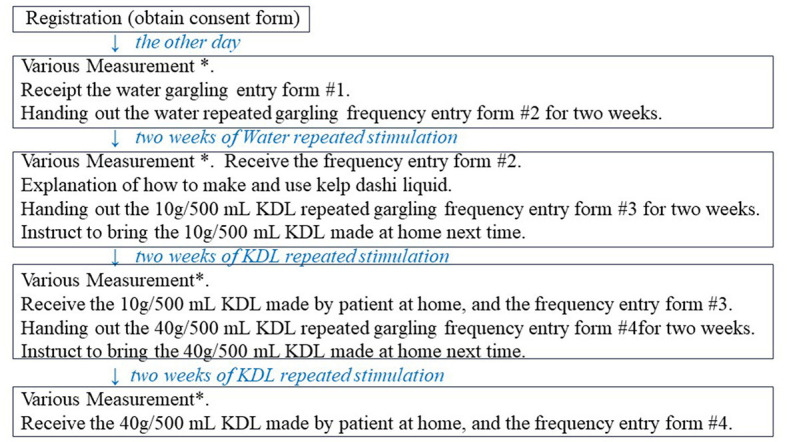
Overview of this study procedure. ^*^Various measurements: measurements of minor salivary flow rate (MF), whole salivary flow rate (WF), recognition threshold (RT) of each of the five basic tastes, and intensity/severity of eating difficulties and other symptoms.

The experimental procedure was performed as shown in Figure 1. Patients determined to have decreased MF via measurements were explained the details of the study, and they provided informed consent. On another day after registration, we measured the MF, WF, and recognition thresholds (RTs) for the five basic tastes—sweet, salty, sour, bitter, and umami—and assessed the intensity/severity of various dry mouth-related symptoms. All patients were given similar instructions, i.e., to keep 25 mL (divided into 2 or 3 times) of water or KDL (10 g/500 mL, or 40 g/500 mL) in the mouth and gargle for 30 s, 10 times daily (example: 3, 4, and 3 times in the morning, afternoon, and evening, respectively) for 2 weeks, savoring the taste thoroughly. Subsequently, patients were given a form to record the number of times they gargled each day and were instructed to bring this record form, along with the KDL that they had made and used, the next day after completing the 2-week gargling stimulation. On the day after each repeated gargle stimulation with water or KDL gargling, we measured the MF, WF, and RT for each of the five basic tastes and assessed the intensity/severity of various dry mouth-related symptoms in the same way as on the day before the research started (Figure 1). The repeated gargling stimulation was performed for 2 weeks, first with water, followed by 10 g/500 mL KDL, and finally with 40 g/500 mL KDL. The patients were instructed to prepare the KDL as follows: finely chop the kelp that you have at home (10 g or 40 g), put it in a plastic bottle with 500 mL of water, and leave it at room temperature overnight. After preparation, it was stored in the refrigerator, and when used, it was returned to room temperature and used for gargling. KDL contains amino acids, such as glutamic acid, which are nutrients that can promote the growth of microorganisms; therefore, KDL spoils easily. To ensure that the participants used KDL safely, pre-prepared KDL was not provided to them; instead, the participants made the KDL at home using a common recipe provided to them. The participants prepared fresh KDL regularly every 2 days at home using this recipe.

### 2.3 Measurements of MF and WF

The MF was measured using an electronic device that measures the impedance of a fine filter paper (27). The patient's cheeks and lips were held with a cheek retractor; the accumulated saliva was absorbed with a roll wattle after air-drying the lower lip; a 10 × 10 mm filter paper (Toyo Roshi Kaisha, Ltd., Tokyo, Japan) was placed on the center of the lip, 5 mm from the bottom of the oral vestibule; and the saliva secreted from minor salivary glands was absorbed for 1 min. The filter paper was fixed to the central part of the electrode, and the displayed electrical potential difference value was read. The MF was obtained using the calibration curve and conversion formula from the potential difference value of the filter paper that absorbed the saliva. A decreased MF was defined as a cut-off value of < 1.0 μL/cm^2^/min, based on our previous research (28).

The WF was measured using the gum test (29), in which the patients chewed gum and spit out the saliva secreted into a plastic tube for 10 min. A decreased WF was defined as a flow rate of < 10 mL/10 min (29).

The MF and WF were measured in all patients 1 h after meals and tooth brushing.

### 2.4 Measurements of the RTs of the five basic tastes

The RTs of the four basic tastes—sweet, salty, sour, and bitter—were measured using Taste Disks^®^ (Sanwa Chemical Co., Ltd., Nagoya, Japan) that consisted of five concentrations of the test solutions for each taste quality (30–32). The concentrations of the four basic tastes were as follows:

i) Sweet (sucrose): 8.7 mM (No. 1:S1), 73.0 mM (No. 2: S2), 292.1 mM
(No. 3: S3), 584.2 mM (No. 4: S4), and 2,337.1 mM (No. 5: S5).
ii) Salty (NaCl): 51.3 mM (No. 1:N1), 213.8 mM (No. 2: N2), 855.5 mM
(No. 3: N3), 1,711.1 mM (No. 4: N4), and 3,422.3 mM (No. 5: N5).
iii) Sour (tartaric acid): 1.3 mM (No. 1:T1), 13.3 mM (No. 2: T2), 133.2 mM
(No. 3: T3), 266.5 mM (No. 4: T4), and 533.0 mM (No. 5: T5).
iv) Bitter (quinine): 0.03 mM (No. 1:Q1), 0.5 mM (No. 2: Q2), 2.5 mM
(No. 3: Q3), 12.6 mM (No. 4: Q4), and 100.7 mM (No. 5: Q5).

In this study, for each of the four basic tastes, No. 6 was used when the taste could not be determined at the concentration of No.5.

To measure the RT of umami, an aqueous solution of monosodium glutamate (MSG) was used (33), which included six concentrations of the test solution: 1 mM (No. 1:G1), 5 mM (No. 2: G2), 10 mM (No. 3:G3), 50 mM (No. 4: G4), 100 mM (No. 5:G5), and 200 mM (No. 6: G6). In this study for the umami taste, No. 7 was used when the taste could not be determined at the concentration of No. 6.

The round filter paper disc (5 mm in diameter) soaked in each taste solution was placed for 3 s on the anterior tongue. Subsequently, the paper disc was removed, and the participants were asked if they perceived any taste and to specify the type of taste they perceived. The RT was identified based on the solution number of the lowest concentration of taste solution at which the patients could correctly recognize the taste quality. The measurement was started from the lowest concentration of each taste solution until the RT was identified. To avoid interference between tastes, the patients rinsed their mouths with water several times until no previous taste remained. The order of the taste test was random except for the bitter taste test, which was performed at the end because of the unpleasantness of the taste. All patients were asked to avoid eating or drinking (except water), smoking, and brushing their teeth for at least 2 h before testing.

The diagnostic criteria of taste tests for sweet, salty, sour, and bitter are: number 1 is hypergeusia, number 2 and 3 are normal tastes, number 4 is a low degree of hypogeusia, number 5 is moderate hypogeusia, and number 6 is a high degree of hypogeusia (31).

The diagnostic criteria of the taste test for umami in the taste tests of the anterior tongue area are: number 1 is hypergeusia; number 2, 3, and 4 are normal tastes; number 5 is a low degree of hypogeusia; number 6 is moderate hypogeusia; and number 7 is a high degree of hypogeusia (33).

### 2.5 Measurements of the intensity/severity of eating difficulties and other symptoms

At the beginning of this experiment, the patients were asked if they had any eating difficulties and other symptoms, such as difficulty eating dry foods, difficulty in swallowing, taste impairment, continuous dry mouth sensation during the day, dry mouth sensation at night, difficulty in speaking, and burning sensation in the oral mucosa. For the assessment of the intensity/severity of eating and other difficulties, we used an originally arranged Visual Analog Scale (VAS) (34–36) that consisted of a straight line with the endpoints defining extreme limits, such as “no symptom (such as pain) at all” and “symptom (such as pain) as bad as it could be.”

A line length of 10 or 15 cm showed the smallest measurement error and was most convenient for respondents, as has been previously reported (37). Therefore, we adopted a VAS of 10 cm in length. The patients were asked to mark their symptom intensity/severity level on the line between the two endpoints. The distance (mm) from the left edge (endpoint) to the site marked by the patient was defined as the VAS value.

### 2.6 Quantification of the amounts of various amino acids in the KDL made by patients

The KDL brought by each patient was immediately frozen and sent to the Okinawa Prefectural Health Biotechnology Research and Development Center, where the free amino acid components, such as glutamic acid, aspartic acid, serine, glycine, arginine, alanine, tyrosine, valine, phenylalanine, lysine, proline, threonine, cystine, methionine, isoleucine, leucine, tryptophan, and histidine, were analyzed and quantified. The sample was analyzed after adding three times the volume of 99.5% ethanol and filtering through a 0.2 μm filter. Highly concentrated samples were diluted with 0.1 N hydrochloric acid, filtered in the same way, and analyzed as a sample solution. The standard solution was an amino acid-mixed standard solution of type H and L-tryptophan (Wako Pure Chemical Industries, Ltd.), and the concentrations were prepared at five points in the range of 2 to 5 μmol/L and analyzed. The measurement was performed using an ultra-high-performance liquid chromatograph, the Nexere X2 series (Shimadzu Corporation, Japan), by an automatic pre-column derivation method.

### 2.7 Statistical analysis

The comparison of the prevalence of hypogeusis, eating difficulties, and other symptoms between the two groups was statistically calculated using Pearson's χ^2^ test or ^b^ Fisher's exact test, depending on the variables and their normality. The Shapiro-Wilk test was used to confirm the normality. Comparisons of the changes in the salivary flow rate, in the RTs of each of the five tastes, and the intensities of the various symptoms induced before and after repeated stimulation with water or KDL were performed using the paired *t*-test. We corrected p-values to adjust multiple comparisons based on the Bonferroni correction. Spearman's rank correlation was used for the analysis of the correlation between the amount of glutamic acid in KDL and the change in the amount of saliva due to KDL stimulation. The statistical significance was set at p < 0.05. All statistical analyses were performed using IBM's Statistical Package for the Social Sciences (SPSS), Version 29.01.

## 3 Results

### 3.1 Taste loss and saliva decrease

#### 3.1.1 Characteristics of patients

Participants were meticulously selected for this study; seven patients withdrew due to poor physical condition, six patients dropped out without permission, and six patients used KDL prepared with an inappropriate method, resulting in the inclusion of 35 patients in the analysis. Most of the patients were women (94.2%), aged 72.31 ± 10.61 years. The MF in the lower lip was low in all patients. Patients were divided into two groups depending on whether whole saliva secretion was normal or decreased: 14 patients had a normal whole saliva secretion flow rate (NWS-LMS group, with only dry mouth sensation), and 21 patients had a low whole saliva secretion flow rate (LWS-LMS group, with hyposalivation). Table 1 shows the overall characteristics of the patients in the two groups.

**Table 1:** Characteristics of patients.

**Characteristic**	**NWS-LMS group**	**LWS-LMS group**	***p*-value**
	**(with only dry mouth sensation)**	**(with hyposalivation)**	
	**(*n* = 14)**	**(*n* = 21)**	
**Age**			
Range	44–86	44–86	
Means ± SD	72.9 ± 11.65	72.00 ± 10.15	0.21^a^
	*N* (%)	*N* (%)	
**Sex**			
Female	13 (92.8)	20 (95.2)	0.647 ^d^
Male	1 (8.2)	1 (4.8)	
**History of systemic disease**			
Sjögren's syndrome (SS)	1 (7.1)	8 (38.1)	0.045^d^
Depression/Anxiety disorder (DP/AN)	6 (42.8)	10 (47.6)	0.782^c^
Diabetes mellitus (DM)	1 (7.1)	2 (9.5)	0.652^d^
SS and/or DP/AN and/or DM	1 (7.1)	8 (38.1)	0.045^d^
Other disease (HT, CKD, BD, GID)	8 (57.1)	11 (52.3)	0.528^c^
**Smoking**			
Smoking	1 ( 7.1)	1 (4.7)	0.647^d^
No smoking	13 (92.9)	20 (95.3)	
**Medication**			
Range	0–10	0–10	
Number of types	4.5 ± 3.0	5.0 ± 3.3	0.654^b^

HT, hypertension; CKD, chronic kidney disease; BD, bone disease; GID, gastrointestinal disease.

p-value from ^a^unpaired t-test, ^b^Welch test, ^c^Pearson's χ^2^ test, and ^d^Fisher's exact test.

No significant differences were observed in age (*p* = 0.211), sex (*p* = 0.647), number of medications used (*p* = 0.654), or frequency of smoking (*p* = 0.647) between the two groups. The prevalence of Sjögren's syndrome was significantly higher in the LWS-LMS group than in the NWS-LMS group (*p* = 0.045). There was no significant difference in the prevalence of anxiety/depression (*p* = 0.782) or diabetes (*p* = 0.652) between the two groups. The LWS-LMS group had a significantly larger proportion of patients with multiple dry mouth-related diseases mentioned above than the NWS-LMS group (*p* = 0.045). On the other hand, there was no significant difference in the rate of having non-dry mouth-related diseases, such as hypertension, kidney disease, osteoporosis, and gastroenteritis, between the two groups (*p* = 0.528).

#### 3.1.2 Prevalence of taste loss (hypogeusia) in patients with a salivary decrease or dry mouth sensation

Table 2 shows the prevalence of taste loss (hypogeusia) in patients with hyposalivation. Umami-taste loss (hypogeusia) was notably high, affecting approximately 50% of individuals in both groups, surpassing the prevalence of other taste losses. Although hypogeusia for each of the taste qualities was more prevalent in the LWS-LMS group with hyposalivation than in the NWS-LMS group with only dry mouth sensation, there was no significant difference in the prevalence of hypogeusia for the four taste qualities:salty (*p* = 0.652), sour (*p* = 0.209), bitter (*p* = 0.130), and umami (*p* = 0.581) between the NWS-LMS and LWS-LMS groups.

**Table 2:** Prevalence of hypogeusia (taste loss) on each taste quality revealed by the taste test.

**Hypogeusia**	**NWS-LMS group**	**LWS-LMS group**	***P*-value**
	**(with only dry mouth sensation group)**	**(with hyposalivation group)**	
	**(*n* = 14)**	**(*n* = 21)**	
	***N* (%)**	***N* (%)**	
Hypogeusia for sweet	0 ( 0)	5 (23.8)	
Hypogeusia for salty	1 (7.1)	2 (9.5)	0.652^b^
Hypogeusia for sour	1 (7.1)	5 (23.8)	0.209^b^
Hypogeusia for bitter	1 (7.1)	6 (28.5)	0.130^b^
Hypogeusia for umami	8 (57.1)	10 (47.6)	0.581^a^

The criteria for hypogeusia for sweet, salty, sour, and bitter are a recognition threshold of ≥4.

The criteria for hypogeusia for umami in the anterior tongue area are a recognition threshold of 7.

p-value from ^a^Pearson's χ^2^ test and ^b^Fisher's exact test.

#### 3.1.3 Prevalence of eating difficulties and so on in patients with salivary decrease or dry mouth sensation

Table 3 shows the prevalence of eating difficulties and other symptoms in patients with hyposalivation, or dry mouth sensation. The rates of eating difficulties, such as difficulty in eating dry foods, difficulty in swallowing, and taste impairment, were higher in patients. There were no significant differences in each symptom between the NWS-LMS and LWS-LMS groups: dry mouth at night (NWS-LMS group with only dry mouth sensation, 84.6%; LWS-LMS group with hyposalivation, 84.2%; *p* =0.602), difficulty in speaking (NWS-LMS group, 61.5%; LWS-LMS group, 57.9%; *p* =0.837), difficulty in eating dry foods (NWS-LMS group, 69.2%; LWS-LMS group, 84.2%; *p* =0.281), difficulty in swallowing (NWS-LMS group, 69.2%; LWS-LMS group, 78.9%; *p* =0.431), burning sensation in the oral mucosa (NWS-LMS group, 84.6%; LWS-LMS group, 89.4%; *p* =0.542), and taste impairment (NWS-LMS group, 76.9%; LWS-LMS group, 63.2%; *p* = 0.335).

**Table 3:** Prevalence of eating difficulties and other symptoms in patients.

**Subjective symptoms present**	**NWS-LMS group**	**LWS-LMS group**	***P*-value**
	**(with only dry mouth sensation group)**	**(with hyposalivation group)**	
	**(*n* = 13)**	**(*n* = 19)**	
	***N* (%)**	***N* (%)**	
Continuous dry mouth sensation during the day	13 (100)	19 (100)	
Dry mouth sensation at night	11 (84.6)	16 (84.2)	0.602^b^
Difficulty in speaking	8 (61.5)	11 (57.8)	0.837^a^
Difficulty in eating dry foods	9 (69.2)	16 (84.2)	0.281^b^
Difficulty in swallowing	9 (69.2)	15 (78.9)	0.413^b^
Burning sensation in the oral mucosa	11 (84.6)	17 (89.4)	0.542^b^
Taste impairment	10 (76.9)	12 (63.1)	0.335^b^

p-value from ^a^Pearson's χ^2^ test and ^b^Fisher's exact test.

### 3.2 Effect of repeated stimulation with the KDL gargling on saliva secretion

Although the study started with 14 and 21 patients in the NWS-LMS and LWS-LMS groups, respectively, only eight and 14 patients in the NWS-LMS and LWS-LMS groups, respectively, were able to complete 2 weeks of the three types of repeated stimulations: the first with water, the second with 10 g/500 mL KDL, and the third with 40 g/500 mL KDL.

#### 3.2.1 Changes in MF in the lower lip

Tables 4A, B show the changes in MF before and after repeated stimulation with water and KDL gargling.

**Table 4A:** Change in the minor salivary flow rate (MF) in the NWS-LMS group induced by RG stimulation^*^ first with water and then with kelp dashi liquid (KDL).

**Minor salivary flow rates (μL/cm^2^/min)**	Before RG stimulation^*^	After RG stimulation^*^	**Difference**	***P*-value^†^**
	**Average** ±**SD**	**Average** ±**SD**	**(95% CI)**	
Water (*n* = 8)	0.003 ± 0.004	0.005 ± 0.006	0.00 (0.00, 0.01)	0.902
10 g/500 mL KDL (*n* = 8)	0.005 ± 0.006	0.060 ± 0.050	0.05 (0.02, 0.09)	0.028
40 g/500 mL KDL (*n* = 8)	0.060 ± 0.050	2.533 ± 2.429	2.47 (0.78, 4.16)	0.048

^*^ 2 weeks of repeated gargling stimulation.

^†^ Bonferroni's corrected p-value.

Criteria for hyposalivation: MS < 1.0 μL/cm^2^/min.

KDL, kelp dashi liquid; SD, standard deviation.

**Table 4B:** Change in the minor salivary flow rate (MF) in the LWS-LMS group induced by RG stimulation^*^ first with water and then with kelp dashi liquid (KDL).

**Minor salivary flow rates (μL/cm^2^/min)**	Before RG stimulation^*^	After RG stimulation^*^	**Difference**	***P*-value^†^**
	**Average** ±**SD**	**Average** ±**SD**	**(95% CI)**	
Water (*n* = 14)	0.018 ± 0.026	0.022 ± 0.042	0.00 (−0.01, 0.01)	0.862
10 g/500 mL KDL (*n* = 14)	0.022 ± 0.042	0.877 ± 1.134	0.86 (0.26, 1.45)	0.029
40 g/500 mL KDL (*n* = 14)	0.877 ± 1.134	2.345 ± 2.382	1.47 (0.60, 2.33)	0.011

^*^ 2 weeks of repeated gargling stimulation.

^†^ Bonferroni's corrected p-value.

Criteria for hyposalivation: MS < 1.0 μL/cm^2^/min.

KDL, kelp dashi liquid; SD, standard deviation.

No significant differences were observed in MF after repeated gargling with water in the two groups (NWS-LMS group, *p* = 0.902; LWS-LMS group, *p* = 0.862) (Tables 4A, B).

Repeated stimulation with gargling with 10 g/500 mL KDL for 2 weeks resulted in a significant increase in MF on the day after the stimulation ended in both groups (NWS-LMS group, *p* = 0.028; LWS-LMS group, *p* = 0.029). Moreover, significant MF increases induced by repeated gargling with 40 g/500 mL KDL were observed on the day after the stimulation ended in both groups (NWS-LMS group, *p* = 0.048; LWS-LMS group, *p* = 0.011) (Tables 4A, B).

**Figure 2:**
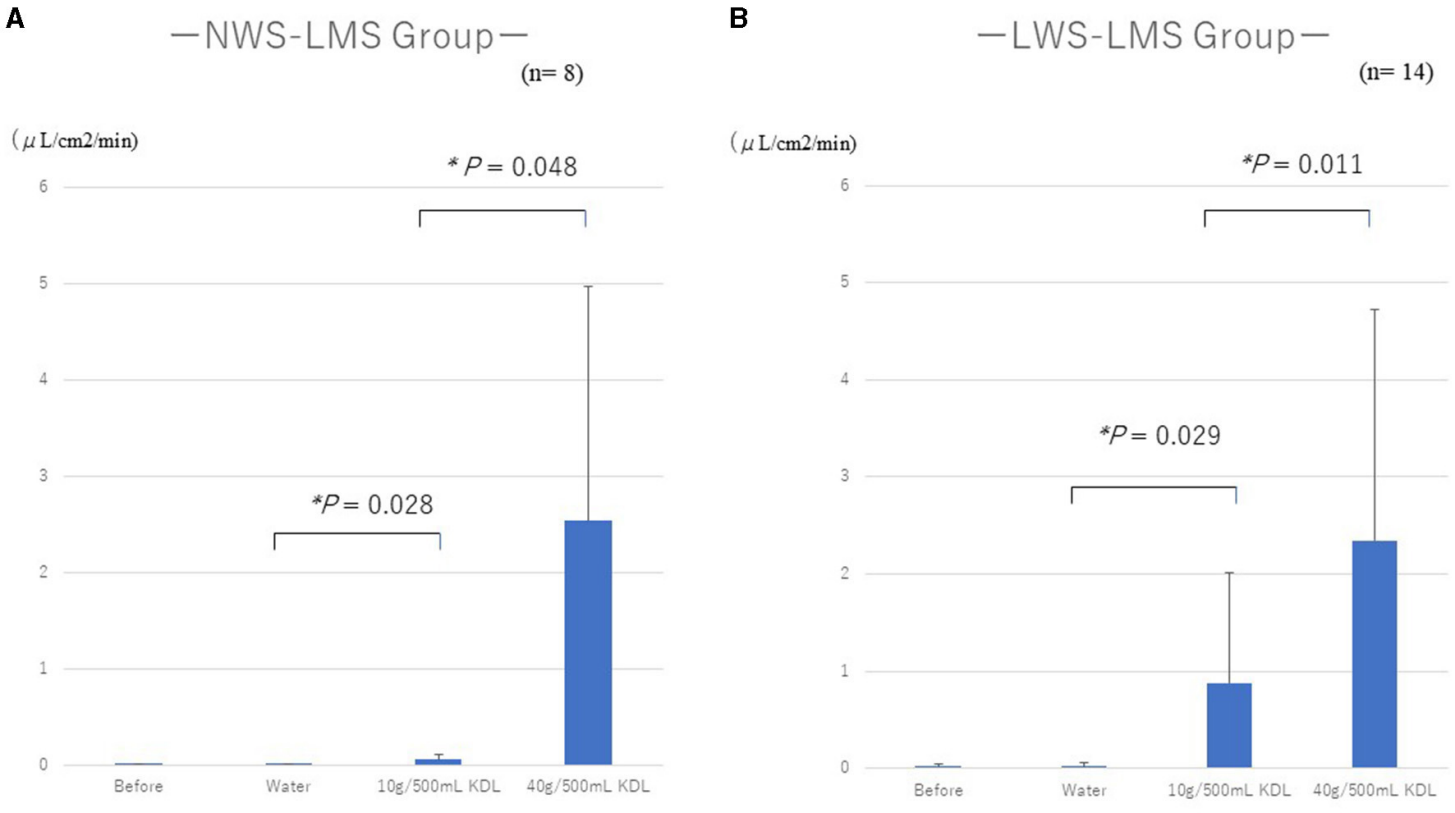
Changes in the minor salivary flow rate (MF) following repeated stimulation with water or KDL gargling in the NWS-LMS group **(A)** and LWS-LMS group **(B)** groups. KDL, kelp dashi liquid; NWS-LMS group, normal whole saliva secretion flow rate with low minor salivary gland secretion flow rate group; LWS-LMS group, low whole saliva secretion flow rate with low minor salivary gland secretion flow rate group.

In both the NWS-LMS and LWS-LMS groups, the MF increases after repeated gargling with 40 g/500 mL KDL were significantly greater than those with 10 g/500 mL KDL (NWS-LMS group, *p* = 0.048; LWS-LMS group, *p* = 0.011) (Figure 2).

#### 3.2.2 Changes in WF

Tables 5A, B show the changes in WF before and after repeated stimulation with water or KDL gargling. No significant differences were observed in WF after repeated gargling with water in the two groups (NWS-LMS group, *p* = 1.000; LWS-LMS group, *p* = 1.000) (Tables 5A, B).

**Table 5A:** Change in the whole salivary flow rate (WF) in the NWS-LMS group induced by RG stimulation^*^ first with water and then with kelp dashi liquid (KDL).

**Whole salivary flow rates (mL/10 min)**	Before RG stimulation^*^	After RG stimulation^*^	**Difference**	***P*-value^†^**
	**Average** ±**SD**	**Average** ±**SD**	**(95% CI)**	
Water (*n* = 8)	12.50 ± 2.71	12.45 ± 2.97	−0.05 (−0.25, 0.15)	1.000
10 g/500 mL KDL (*n* = 8)	12.45 ± 2.97	13.15 ± 3.35	0.70 (−1.14, 2.54)	0.960
40 g/500 mL KDL (*n* = 8)	13.15 ± 3.35	13.80 ± 4.50	0.65 (−0.86, 2.16)	0.852

^*^ 2 weeks of repeated gargling stimulation.

^†^ Bonferroni's corrected p-value.

Criteria for hyposalivation in WF < 10 mL/10 min.

KDL, kelp dashi liquid; SD, standard deviation.

**Table 5B:** Change in the whole salivary flow rate (WF) in the LWS-LMS group induced by RG stimulation^*^ first with water and then with kelp dashi liquid (KDL).

**Whole salivary flow rates (mL/10 min)**	Before RG stimulation^*^	After RG stimulation^*^	**Difference**	***P*-value^†^**
	**Average** ±**SD**	**Average** ±**SD**	**(95% CI)**	
Water (n = 14)	3.85 ± 2.40	3.80 ± 2.48	−0.06 (−0.26, 0.14)	1.000
10 g/500 mL KDL (*n* = 14)	3.80 ± 2.48	4.30 ± 2.92	0.50 (0.13, 0.87)	0.041
40 g/500 mL KDL (*n* = 14)	4.30 ± 2.92	4.65 ± 2.86	0.35 (0.20, 0.65)	0.005

^*^ 2 weeks of repeated gargling stimulation.

^†^Bonferroni's corrected p-value.

Criteria for hyposalivation in WF < 10 mL/10 min.

KDL, kelp dashi liquid; SD, standard deviation.

In the NWS-LMS group, no significant changes in WF were observed after stimulation with repeated gargling with both concentrations of KDL (10 g/500 mL, *p* = 0.960; 40 g/500 mL, *p* = 0.852) (Table 5A).

In the LWS-LMS group, significant WF increases induced by repeated gargling with both concentrations of KDL were observed on the day after the stimulation ended (10 g/500 mL, *p* = 0.041; 40 g/500 mL, *p* = 0.005) (Table 5B).

**Figure 3:**
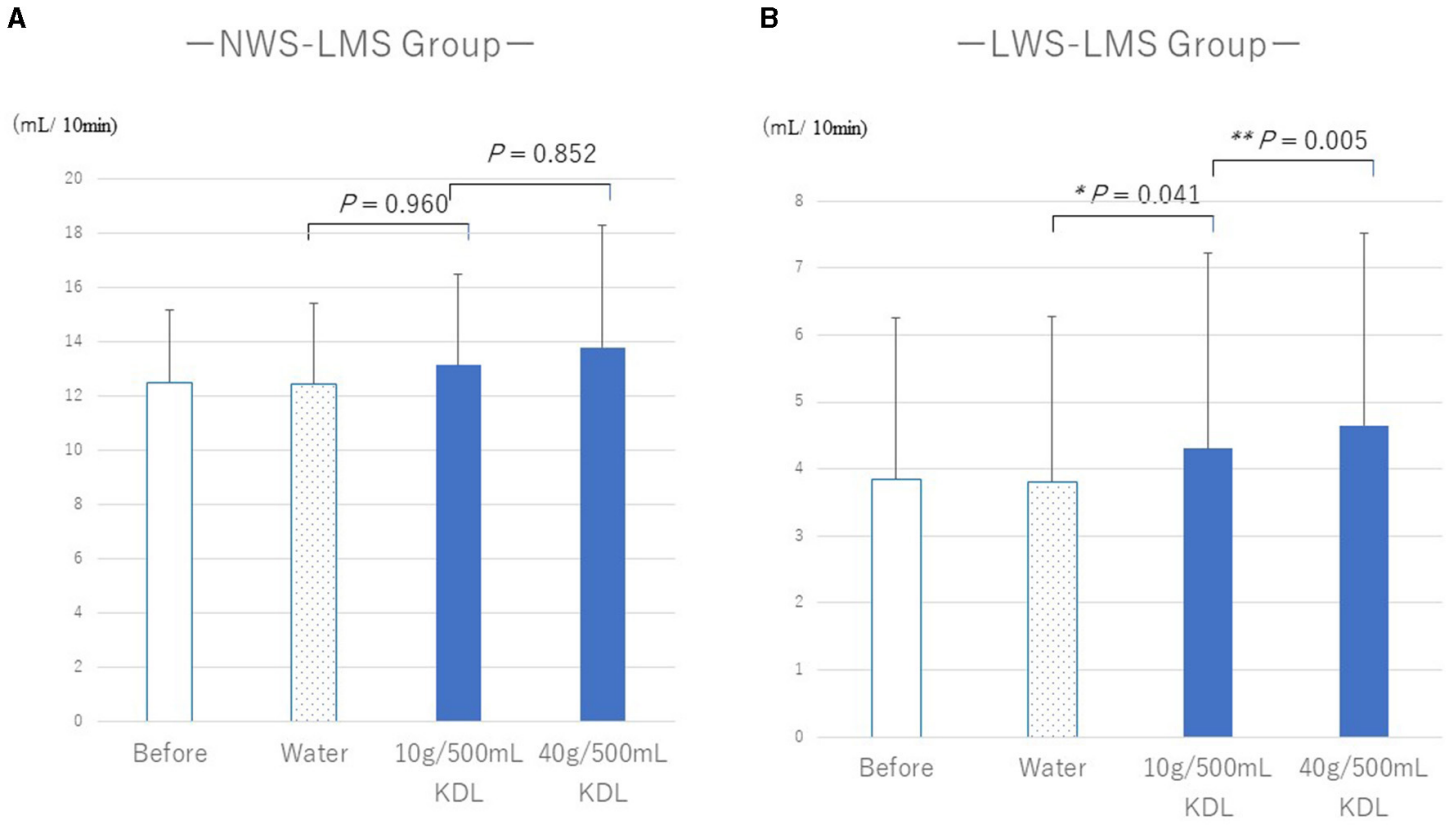
Changes in the whole salivary flow rate (WF) following repeated gargling stimulation with water or KDL gargling in the NWS-LMS **(A)** and LWS-LMS **(B)** groups. KDL, kelp dashi liquid; NWS-LMS group, normal whole saliva secretion flow rate with low minor salivary gland secretion flow rate group; LWS-LMS group, low whole saliva secretion flow rate with low minor salivary gland secretion flow rate group.

In the LWS-LMS groups, the WF increases after repeated gargling with 40 g/500 mL KDL were significantly greater than those with 10 g/500 mL KDL (*p* = 0.005) (Figure 3).

There were no Sjögren's syndrome (SS) patients in the NWS-LMS group who were able to complete three consecutive stimulations; however, there were five SS out of 14 patients in the LWS-LMS group. In the LWS-LMS group, there was no difference in the amount of change in WF and MF induced by repeated stimulation with 40 g/500 mL KDL gargling between the Sjögren's syndrome (SS) patient group and the non-Sjögren's syndrome (NS) patient group. The amount of change in MF was 1.365 ±0.822 μL/cm^2^/min in the SS patients group (*n* = 5), 1.524 ± 2.026 μL/cm^2^/min in the NS patients group (*n* = 9); *p* = 1.000; and the amount of change in WF was 0.400 ±0.430 ml/10 min in the SS patients group (*n* = 5), 0.322 ± 0.327 ml/10 min in the NS patients group (*n* = 9); *p* = 1.000.

### 3.3 Amino acids in KDL and an increase in saliva

#### 3.3.1 Quantification of various amino acids in KDL made by each patient

**Figure 4:**
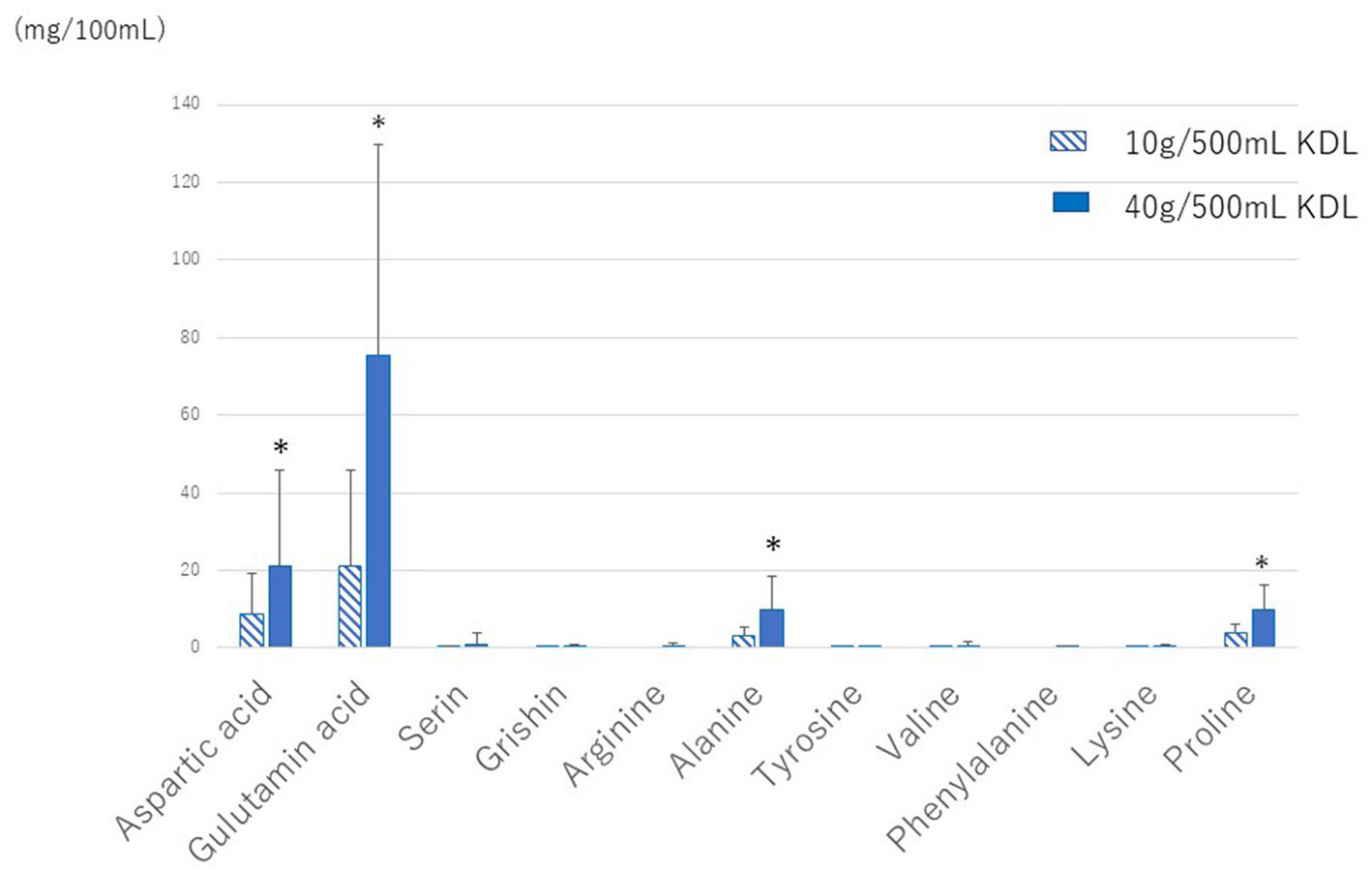
Amounts of various amino acids in KDL made by each patient. KDL, kelp dashi liquid. ^*^ 2 weeks of repeated gargling stimulation.

The amounts of the various amino acids in the KDL made by each patient were quantitatively analyzed. Glutamic acid was the most abundant, followed by alginic acid, while other amino acids were present in very small amounts (Figure 4). The amounts of glutamic acid and other amino acids were larger in 40 g/500 mL KDL than in 10 g/500 mL KDL. There was no difference in the amount of glutamic acid in the KDL made by each patient between the NWS-LMS and LWS-LMS groups (10 g/500 mL KDL, *p* = 0.428; 40 g/500 mL KDL, *p* = 0.745) (Table 6).

**Table 6:** Amount of glutamic acid in kelp dashi liquid (KDL) made by patients.

**Glutamic acid**	**NWS-LMS group**	**LWS-LMS group**	***P*-value**
**(mg /100 mL)**	**Average** ±**SD**	**Average** ±**SD**	
10 g/500 mL KDL	25.56 ± 14.71	32.73 ± 26.83	0.428^b^
40 g/500 mL KDL	81.27 ± 43.91	76.33 ± 36.16	0.745^a^

p-value from ^a^unpaired t-test and ^b^Welch test.

KDL, Kelp dashi liquid; SD, standard deviation.

#### 3.3.2 Correlation between the changes in MF or WF and the amount of glutamic acid

Spearman's correlation analysis showed that the increase in MF following repeated stimulation with KDL gargling was correlated with the amount of glutamic acid in KDL in both groups (NWS-LMS group, r = 0.711, *p* < 0.01; LWS-LMS group, r = 0.490, *p* < 0.01). On the other hand, the increase in WF following repeated stimulation with KDL gargling was not correlated with the amount of glutamic acid in KDL in both groups (NWS-LMS group, r = 0.361; LWS-LMS group, r = 0.072).

### 3.4 Effects of repeated stimulation with KDL gargling on the RTs of the five basic tastes

The repeated stimulation with water gargling did not change the RT in both groups.

Repeated stimulation with KDL gargling significantly reduced the RT for umami, returning the value to normal in both groups: NWS-LMS group: 10 g/500 mL KDL, *p* < 0.0001; 40 g/500 mL KDL, *p* < 0.0001 (Table 7A); LWS-LMS group: 10 g/500 mL KDL, *p* < 0.0001; 40 g/500 mL KDL, *p* < 0.0001 (Table 7B).

**Table 7A:** Change in the recognition threshold (RT) of each taste in the NWS-LMS group induced by RG stimulation^*^ with water and with kelp dashi liquid (KDL).

**Recognition threshold (RT)**	Before RG stimulation^*^	After RG stimulation^*^	**Difference**	***P*-value^†^**
	**Average** ±**SD**	**Average** ±**SD**	**(95% CI)**	
Sweet	(*n* = 8)	(*n* = 8)		
Water	2.7 ± 0.5	2,6 ± 0.5	−0.13 (−0.37, 0.12)	1.000
10 g/500 mL KDL	2.6 ± 0.5	2.3 ± 0.5	−0.38 (−0.72, −0.02)	0.398
40 g/500 mL KDL	2.3 ± 0.5	1.9 ± 0.7	−0.38 (−0.72, −0.02)	0.398
Salty	(*n* = 8)	(*n* = 8)		
Water	2.5 ± 0.8	2.4 ± 0.7	−0.13 (−0.37, 0.12)	1.000
10 g/500 mL KDL	2.4 ± 0.7	2.3 ± 0.5	−0.13 (−0.37, 0.12)	1.000
40 g/500 mL KDL	2.3 ± 0.5	1.9 ± 0.7	−0.38 (−0.89, 0.14)	0.985
Sour	(*n* = 8)	(*n* = 8)		
Water	2.7 ± 0.7	2.6 ± 0.7	−0.13 (−0.37, 0.12)	1.000
10 g/500 mL KDL	2.6 ± 0.7	2,6 ± 0.8	0.00 (0.00, 0.00)	1.000
40 g/500 mL KDL	2.6 ± 0.8	2.3 ± 0.5	−0.25 (−0.57, 0.07)	0.852
Bitter	(*n* = 8)	(*n* = 8)		
Water	2.7 ± 1.0	2.6 ± 0.8	−0.13 (−0.57, 0.32)	1.000
10 g/500 mL KDL	2.6 ± 0.8	2.5 ± 0.7	−0.13 (−0.37, 0.12)	1.000
40 g/500 mL KDL	2.5 ± 0.7	2.1 ± 0.4	−0.38 (−0.73, −0.02)	0.398
Umami	(*n* =8)	(*n* = 8)		
Water	6.5 ± 0.7	6.3 ± 0.8	−0.13 (−0.37, 0.12)	1.000
10 g/500 mL KDL	6.3 ± 0.8	4.7 ± 0.9	−1.63 (−1.98, −1.27)	< 0.0001
40g/500 mL KDL	4.7 ± 0.9	2.4 ± 0.8	−2.38 (−2.73, −2.02)	< 0.0001

^*^ 2 weeks of repeated gargling stimulation.

Criteria for hypogeusia for sweet, salty, sour, and bitter: recognition threshold (RT) ≥4.

Criteria for hypogeusia for umami in the anterior tongue area: recognition threshold (RT) number of 7.

^†^ Bonferroni's corrected p-value.

KDL, kelp dashi liquid; SD, standard deviation.

**Table 7B:** Change in the recognition threshold (RT) of each taste in the LWS-LMS group induced by RG stimulation^*^ with water and with kelp dashi liquid (KDL.

**Recognition threshold (RT)**	Before RG stimulation^*^	After RG stimulation^*^	**Difference**	***P*-value^†^**
	**Average** ±**SD**	**Average** ±**SD**	**(95% CI)**	
Sweet	(*n* = 14)	(*n* = 14)		
Water	3.1 ± 0.8	3.0 ± 0.9	−0.07 (−0.22, 0.07)	1.000
10 g/500 mL KDL	3.0 ± 0.9	2.7 ± 0.5	−0.36 (−0.62, −0.10)	0.093
40 g/500 mL KDL	2.7 ± 0.5	2.1 ± 0.3	−0.64 (−0.90, −0.38)	0.002
Salty	(*n* = 14)	(*n* = 14)		
Water	2.5 ± 0.7	2.4 ± 0.6	−0.14 (−0.33, 0.05)	0.824
10 g/500 mL KDL	2.4 ± 0.6	2.2 ± 0.6	−0.21 (−0.52, 0.09)	0.947
40 g/500 mL KDL	2.2 ± 0.6	1.7 ± 0.5	−0.50 (−0.77, −0.23)	0.016
Sour	(*n* = 14)	(*n* = 14)		
Water	3.0 ± 0.8	2.9 ± 0.7	−0.07 (−0.32, 0.18)	1.000
10 g/500 mL KDL	2.9 ± 0.7	2,6 ± 0.5	−0.36 (−0.62, −0.10)	0.093
40 g/500 mL KDL	2.6 ± 0.5	2.1 ± 0.3	−0.50 (−0.77, −0.23)	0.016
Bitter	(*n* =14)	(*n* = 14)		
Water	3.0 ± 0.8	2.9 ± 0.7	−0.07 (−0.21, 0.07)	1.000
10 g/500 mL KDL	2.9 ± 0.7	2.6 ± 0.5	−0.36 (−0.62, −0.10)	0.093
40 g/500 mL KDL	2.6 ± 0.5	2.1 ± 0.4	−0.43 (−0.70, −0.16)	0.040
Umami	(*n* =14)	(*n* = 14)		
Water	6.0 ± 1.2	6.0 ± 1.1	0.00 (−0.21, 0.21)	1.000
10 g/500 mL KDL	6.0 ± 1.1	4.7 ± 1.1	−1.29 (−1.67, −0.91)	< 0.0001
40 g/500 mL KDL	4.7 ± 1.1	3.1 ± 0.8	−1.57 (−2.10, −1.04)	< 0.0001

^*^ 2 weeks of repeated gargling stimulation.

Criteria for hypogeusia for sweet, salty, sour, and bitter: recognition threshold (RT) ≥4.

Criteria for hypogeusia for umami in the anterior tongue area: recognition threshold (RT) number of 7.

^†^Bonferroni's corrected p-value.

KDL, kelp dashi liquid; SD, standard deviation.

In the NWS-LMS group, there was no difference in the RT of the four basic tastes, i.e., sweet, salty, sour, and bitter, with almost no hypogeusia except for the umami taste (Table 7A).

In the LWS-LMS group, repeated stimulation with 40 g/500 mL KDL gargling significantly reduced each taste's RT (sweet: 10 g/500 mL KDL, *p* = 0.093; 40 g/500 mL KDL, *p* = 0.002, salty: 10 g/500 mL KDL, *p* = 0.947; 40 g/500 mL KDL, *p* = 0.016, sour: 10 g/500 mL KDL, *p* = 0.093; 40 g/500 mL KDL, *p* = 0.016, bitter: 10 g/500 mL KDL, *p* = 0.093; 40 g/500 mL KDL, *p* = 0.040) (Table 7B).

### 3.5 Effects of repeated stimulation with KDL gargling on eating difficulties and other symptoms

Depending on the concentration of KDL, repeated stimulation with gargling alleviated eating difficulties and other symptoms. No significant difference was observed in the dry mouth-related symptoms after repeated stimulation with water gargling.

Continuous dry mouth sensation during the day was reduced by repeated stimulation with KDL in both groups: NWS-LMS group: 10 g/500 mL KDL, *p* = 0.050; 40 g/500 mL KDL, *p* < 0.001 (Table 8A); LWS-LMS group: 10 g/500 mL KDL, *p* = 0.009; 40 g/500 mL KDL, *p* = 0.011 (Table 8B).

**Table 8A:** Change in the intensity/severity (VAS) of subject symptoms in the NWS-LMS group. Induced by RG stimulation^*^ with water and with kelp dashi liquid (KDL).

Intensity/severity (VAS) after RG stimulation^*^	Before RG stimulation^*^	After RG stimulation^*^	**Difference**	***P*-value^†^**
	**Average** ±**SD**	**Average** ±**SD**	**(95% CI)**	
Continuous dry mouth feeling during the day	(*n* = 8)	(*n* = 8)		
Water -	100	96.8 ± 4.5	–	-
10 g/500 mL KDL	96.8 ± 4.5	70.6 ± 18.9	−26.3 (−40.0, −12.6)	0.050
40 g/500 mL KDL	70.6 ± 18.9	32.7 ± 16.1	−37.9 (−45.6, −30.1)	< 0.001
Dry mouth feeling at night	(*n* = 8)	(*n* = 8)		
Water	100	100	–	-
10 g/500 mL KDL	100	91.4 ±18.6	−8.6 (−21.6, 4.3)	1.000
40 g/500 mL KDL	91.4 ±18.6	46.2 ±38.5	−45.1 (−70.4, −19.9)	0.069
Difficulty in speaking	(*n* = 8)	(*n* = 8)		
Water	100	100	–	-
10 g/500 mL KDL	100	72.5 ±17.1	−27.5 (−39.4, −15.6)	0.019
40 g/500 mL KDL	72.5 ±17.1	37.5 ± 17.5	−35.0 (−52.0, −18.0)	0.035
Difficulty in eating dry foods	(*n* = 7)	(*n* = 7)		
Water	100	100	–	-
10 g/500 mL KDL	100	90.0 ± 8.1	−10.0 (−16.1, −4.0)	0.124
40 g/500 mL KDL	90.0 ± 8.1	31.4 ± 22.7	−58.6 (−79.3, −37.9)	0.010
Difficulty in swallowing	(n = 8)	(n = 8)		
Water	100	98.9 ± 3.3	–	-
10 g/500 mL KDL	98.9 ± 3.3	72.5 ± 35.9	−26.4 (−52.0, −0.8)	0.584
40 g/500 mL KDL	72.5 ± 35.9	36.2 ± 16.0	−36.3 (−55.1, −17.4)	0.049
Burning sensation of oral mucosa	(*n* = 8)	(*n* = 8)		
Water	100	100	–	-
10 g/500 mL KDL	100	60.0 ± 33.5	−40.0 (−63.2, −16.8)	0.082
40 g/500 mL KDL	60.0 ± 33.5	27.5 ± 24.4	−32.5 (−48.9, −16.1)	0.042
Taste impairment	(*n* = 8)	(*n* = 8)		
Water	100	98.0 ± 6.3	–	-
10 g/500 mL KDL	98.0 ± 6.3	69.0 ± 28.3	−29.0 (−48.9, −9.1)	0.171
40 g/500 mL KDL	69.0 ± 28.3	13.3 ± 15.0	−55.8 (−77.0, −34.5)	0.009

^*^ 2 weeks of repeated gargling stimulation.

^†^Bonferroni's corrected p-value.

KDL, kelp dashi liquid; SD, standard deviation.

**Table 8B:** Changes in the intensity/severity (VAS) of subject symptoms in the LWS-LMS group. Induced by RG stimulation^*^ with water and with kelp dashi liquid (KDL).

Intensity/severity (VAS) after RG stimulation^*^	Before RG stimulation^*^	After RG stimulation^*^	**Difference**	***P*-value^†^**
	**Average** ±**SD**	**Average** ±**SD**	**(95% CI)**	
Continuous dry mouth feeling during the day	(*n* = 14)	(*n* = 14)		
Water	100	98.1 ± 3.8	–	-
10 g/500 mL KDL	98.1 ± 3.8	76.4 ± 20.1	−21.7 (−32.1, −11.3)	0.009
40 g/500 mL KDL	76.4 ± 20.1	55.3 ± 22.2	−21.1 (−32.4, −9.9)	0.011
Dry mouth feeling at night	(*n* = 9)	(*n* = 9)		
Water	100	100	–	-
10 g/500 mL KDL	100	85.0 ±16.9	−15.0 (−26.1, −3.9)	0.204
40 g/500 mL KDL 85.0 ±16.9		82.2 ±18.5	−2.8 (−15.5, 9.9)	1.000
Difficulty in speaking	(n = 11)	(n = 11)		
Water	100	100	–	-
10 g/500 mL KDL	100	96.7 ± 7.1	−3.3 (−7.5, 0.9)	1.000
40 g/500 mL KDL	96.7 ± 7.1	70.9 ± 22.5	−25.8 (−38.0, −13.6)	0.014
Difficulty in eating dry foods	(*n* = 12)	(*n* = 12)		
Water	100	100	–	-
10 g/500 mL KDL	100	85.8 ± 17.8	−14.2 (−24.3, −4.1)	0.131
40 g/500 mL KDL	85.8 ± 17.8	71.0 ± 26.5	−14.8 (−23.3, −6.2)	0.042
Difficulty in swallowing	(*n* = 14)	(*n* = 14)		
Water	100	100	-	-
10 g/500 mL KDL	100	84.1 ± 21.1	−15.9 (−26.9, −4.8)	0.104
40 g/500 mL KDL	84.1 ± 21.1	61.4 ± 30.3	−22.7 (−31.6, −13.8)	0.002
Burning sensation of oral mucosa	(*n* = 14)	(*n* = 14)		
Water	100	99.4 ± 2.4	-	-
10 g/500 mL KDL	99.4 ± 2.4	58.6 ± 32.3	−40.9 (−57.4, −24.3)	0.002
40 g/500 mL KDL	58.6 ± 32.3	32.8 ± 28.5	−25.8 (−39.6, −12.0)	0.020
Taste impairment	(*n* = 12)	(*n* = 12)		
Water	100	99.1 ± 2.9	-	-
10 g/500 mL KDL	99.1 ± 2.9	58.9 ± 30.2	−40.3 (−57.9, −22.6,)	0.007
40 g/500 mL KDL	58.9 ± 30.2	35.0 ± 26.5	−23.9 (−32.6, −15.2)	0.002

^*^ 2 weeks of repeated gargling stimulation.

^†^ Bonferroni's corrected p-value.

KDL, kelp dashi liquid; SD, standard deviation.

In the NWS-LMS group, repeated stimulation with KDL gargling significantly reduced below-subject symptoms: difficulty in speaking: *p* = 0.019,10 g/500 mL KDL; *p* = 0.035, 40 g/500 mL KDL; difficulty in eating dry foods: *p* = 0.010, 40 g/500 mL KDL; difficulty in swallowing: *p* = 0.049, 40 g/500 mL KDL; burning sensation in the oral mucosa: *p* = 0.042, 40 g/500 mL KDL; taste impairment: *p* = 0.009, 40 g/500 mL KDL) (Table 8A).

In the LWS-LMS group, repeated stimulation with KDL gargling significantly reduced below-subject symptoms: difficulty in speaking: *p* = 0.014, 40 g/500 mL KDL; difficulty in eating dry foods: *p* = 0.0042, 40 g/500 mL KDL; difficulty in swallowing: *p* =0.002, 40 g/500 mL KDL; burning sensation in the oral mouth: *p* =0.002,10 g/500 mL KDL; *p* =0.020, 40 g/500 mL KDL; taste impairment: *p* =0.007,10 g/500 mL KDL; *p* =0.002, 40 g/500 mL KDL) (Table 8B). There was no significant difference in dry mouth sensation at night by repeated simulation with KDL in both groups.

Table 9 shows a difference in the effects of KDL stimulation on each subject's symptoms between the two groups. Improvement of VAS values with KDL stimulation for difficulty in speaking (*p* =0.033, 10 g/500 mL KDL; *p* =0.015, 40 g/500 mL KDL), and difficulty in eating dry foods (*p* =0.026, 40 g/500 mL KDL) were more significant in the NWS-LMS group than in the LWS-LMS group (Table 9).

**Table 9:** Comparison of intensity/severity (VAS) change of subjective symptoms by RG stimulation^*^ between NWS-LMS and LWS-LMS groups.

Intensity/severity (VAS) after RG stimulation^*^	**NWS-LMS group**	**LWS-LMS group**	**Difference**	***P*-value^†^**
	**Average** ±**SD**	**Average** ±**SD**	**(95% CI)**	
Continuous dry mouth feeling during the day	(*n* = 8)	(*n* = 14)		
Water	96.8 ± 4.5	98.1 ± 3.8	–	-
10 g/500 mL KDL	70.6 ±18.9	76.4 ±20.1	5.8 (−11.1, 22.7)	1.000
40 g/500 mL KDL	32.7 ±16.1	55.3 ±22.2	22.5 (5.8, 39.3)	0.091
Dry mouth feeling at night	(*n* = 8)	(*n* = 9)		
Water	100	100	–	-
10 g/500 mL KDL	91.4 ±18.6	85.0 ±16.9	−6.4 (−23.4, 10.7)	1.000
40 g/500 mL KDL	46.2 ±38.5	82.2 ±18.5	36.0 (6.7, 65.3)	0.262
Difficulty in speaking	(*n* = 8)	(*n* = 11)		
Water	100	100	–	-
10 g/500 mL KDL	72.5 ±17.1	96.7 ± 7.1	24.2 (11.6, 36.8)	0.033
40 g/500 mL KDL	37.5 ±17.5	70.9 ±22.5	33.4 (15.4, 51.4)	0.015
Difficulty in eating dry foods	(*n* = 7)	(*n* = 12)		
Water	100	100	–	-
10 g/500 mL KDL	90.0 ± 8.1	85.8 ±17.8	−4.2 (−15.9, 7.6)	1.000
40 g/500 mL KDL	31.4 ±22.7	71.0 ±26.5	39.7 (18.0, 61.4)	0.026
Difficulty in swallowing	(*n* = 8)	(*n* = 14)		
Water	98.9 ± 3.3	100	–	-
10 g/500 mL KDL	72.5 ±35.9	84.1 ±21.1	11.6 (−15.6, 38.9)	1.000
40 g/500 mL KDL	36.2 ±16.0	61.4 ±30.3	25.2 (5.8, 44.6)	0.134
Burning sensation of oral mucosa	(*n* = 8)	(*n* = 14)		
Water	100	99.4 ± 2.4	–	-
10 g/500 mL KDL	60.0 ±33.5	58.6 ±32.3	−1.4 (−30.2, 27.3)	1.000
40 g/500 mL KDL	27.5 ±24.4	32.8 ±28.5	5.3 (−17.3, 27.9)	1.000
Taste impairment	(*n* = 8)	(*n* = 12)		
Water	98.0 ± 6.3	99.1 ± 2.9	–	-
10 g/500 mL KDL	69.0 ± 28.3	58.9 ± 30.2	−10.1 (−36.1, 15.9)	1.000
40 g/500 mL KDL	13.3 ± 15.0	35.0 ± 26.5	21.8 (3.5, 40.0)	0.219

^*^ 2 weeks of repeated gargling stimulation.

^†^Bonferroni's corrected p-value.

KDL, kelp dashi liquid; SD, standard deviation.

## 4 Discussion

This study revealed that the umami taste in KDL might contribute to the maintenance of the normal taste sensation and promote healthy eating habits in older adults. First, repeated stimulation with KDL improved taste decline and maintained the normal taste sensation by increasing saliva production in MF and WF in older adults through the umami-taste gustatory reflex. Second, repeated stimulation with KDL alleviated subjective taste impairment and subjective eating and swallowing difficulties.

In this study, the incidence of taste loss in the patients with hyposalivation (LWS-LMS group) was 9.5% for the salty taste, approximately 24% for the sweet and sour taste, and 28.5% for the bitter taste. However, umami-taste loss was the most prevalent, with an incidence of 48% occurring in approximately half of the patients. In the patients with only oral dryness (NWS-LMS group), the incidence of umami-taste loss was 57%, which is similar to that in the patients with hyposalivation patients, although it was 7% for the salty, sour, and bitter tastes. Since taste sensation is associated with WF, hyposalivation, which involves decreased WF, is known to be the most common cause of taste loss in older adults (5). However, the relationship between taste loss and decreased MF has not yet been established. Our results revealed that umami-taste loss is more likely to occur in older people with dry mouths than taste loss of the other four basic taste qualities. Moreover, in particular, umami-taste loss was also observed in older adults with decreased MF and normal WF, which was similar to the finding in patients with hyposalivation. This suggests that umami-taste loss might be more dependent on the decreased MF than on WF.

In this study, repeated stimulation with KDL normalized RTs, improved taste loss for all tastes, including umami-taste, and sustained normal taste sensation in both patients with hyposalivation (LWS-LMS group) and dry mouth sensation (NWS-LMS group).

Umami-taste loss in older people has been reported to be associated with loss of appetite and weight loss. It has been reported that umami-taste loss was observed in 16% of older patients with hypogeusia, who experienced decreased appetite along with weight loss and poor physical condition (38). The umami sensitivity in these patients improved through the treatment, including therapy for increasing saliva; thereafter, these patients regained their appetite and weight, as well as their health (38). In general, dry mouth which causes taste decline and leads to frailty, occurs frequently in older adults. It has also been reported that dry mouth (xerostomia) in older adults is linked to a decline in physical function (39) and the progression of frailty later in life (40). Considering our present findings, it is possible that taste loss caused by dry mouth might also contribute to the onset of frailty in older adults. Taste decline in older adults is less noticeable than vision and hearing loss (41); it is difficult to detect because it occurs gradually. This study shows that umami-taste loss also generally occurs in those who only have dry mouth sensations. Repeated stimulation with KDL gargling could improve and sustainably maintain the taste sensation, especially umami-taste, and lead to maintain their appetite and weight, and it is thought to be effective in preventing frailty in older adults.

Since decreased saliva is a common cause of taste decline in older adults (6, 7), it is necessary to sustain an increase in saliva over a long period of time to improve and maintain the taste sensation. However, it has still been difficult to sustainably increase saliva production to improve taste because older adults are more likely to experience adverse effects caused by medications. The taste-salivary reflex has been studied in an attempt to use it as a remedy to increase saliva production. To date, it has been reported that after an increase induced by a moisturizing gel containing sweet and sour substances, whole saliva returned to normal levels in participants with oral dryness after 10 min (42). In this study, we used umami-rich KDL as a stimulus to induce the umami-taste salivary reflex. Repeated stimulation with KDL increased the decreased MF and WF until the next day after stimulation, implying that the effects lasted at least half a day, the longest period to date. The current results reveal that KDL is effective in continuously increasing saliva production.

KDL contains glutamic acid, which imparts the umami-taste sensation. The KDL made by each patient contained significantly more glutamic acid than other amino acids. As the amount of kelp increased, the glutamic acid in the KDL also increased. The amount of glutamic acid affected the decrease in RT for each taste quality as well as the increase in MF and WF. In other words, it was dependent on the intensity of the umami flavor. We previously reported that in healthy subjects with an average age of 31 years old, the increase in MF induced by the umami taste lasted for more than 25 min after the stimulation ended, whereas the increase in MF induced by the other four basic tastes (sweet, salty, sour, and bitter) returned to the baseline level quickly (24). Moreover, in a comparative study with similar intensities of each of the five taste solutions, the umami-taste increased the MF in the lower, similar to the sour state and significantly more than the sweet, salty, and bitter tastes (25) in healthy volunteers with an average age of 31 years old (31, 33). Considering these previous studies, the umami taste of KDL might have caused a long-lasting increase in MF and WF, even in older patients with xerostomia. The useful effects of KDL on the taste sensation would appear to be based on this finding.

The mechanism behind the long-lasting increase in MF and WF induced by KDL stimulation may depend on the umami aftertaste. The duration of the aftertaste differs depending on each taste quality (43). The sour aftertaste elicited by tartaric acid decreases rapidly after spitting it out, but the umami aftertaste elicited by glutamic acid remains strong and persists even after spitting it out. Umami has unique temporal characteristics, i.e., a long-lasting aftertaste (26).

In this study, the improvement in taste decline induced by KDL stimulation was particularly remarkable in the case of the umami taste. It has been reported that the expression of taste-related genes in the tongue, induced by tongue stimulation with MSG, increased the expression levels of the umami taste-related genes *T1R1* and *T1R3* (44). It is possible that in this study, repeated stimulation with KDL increased the expression of umami taste-related genes *T1R1* and *T1R3* in the tongue, resulting in increased umami sensitivity and improved umami-taste loss.

Older individuals often complain of eating difficulties, including difficulty in swallowing and chewing. In this study, we demonstrated that repeated stimulation with KDL alleviated the subjective difficulty in swallowing and eating dry food not only in older individuals with hyposalivation (LWS-LMS group) but also in those with only dry mouth sensation (NWS-LMS group). In addition, repeated stimulation with KDL increased MF in both groups. These results showed that these subjective eating difficulties occurred when MF was decreased, even if WF was normal. The amount of saliva secreted by the minor salivary glands is extremely small (8% of whole saliva); however, these subjective symptoms might be alleviated by increasing MF via the umami-taste salivary reflex. Until now, subjective difficulty in swallowing and eating has been associated with whole saliva (14, 45), and the relationship with minor gland saliva remains unclear.

In this study, we demonstrated that decreased MF is associated with subjective dry mouth sensations. Previous reports have shown that patients with complaints of dry mouth are less likely to have high MF (46) and that individuals with normal stimulated and resting whole salivary secretion who complain of xerostomia both in the day and night have significantly lower MF (47). Moreover, patients with dry mouth have significantly lower MF than WF compared to healthy subjects (28). In the present study, these KDL-related improvements in subjective eating difficulties were associated with increased MF. Minor gland saliva contains large amounts of mucin and secretory IgA, which contribute to moisturization and are involved in the maintenance of oral mucosal health (17–19). Remarkably, major salivary glands secrete saliva when stimulated, whereas minor gland saliva is constantly secreted, both when stimulated and at rest (15).

The present study is the first to demonstrate that subjective eating difficulties in older individuals occur when MF is decreased, even if WF is normal, and that KDL stimulation alleviates these subjective eating difficulties. KDL improves eating difficulties by increasing MF via the umami-taste salivary reflex, and it could potentially promote healthy daily dietary habits.

Complaints of burning sensation in the oral mucosa are common among oral problems in older individuals. In this study, oral mucosal burning sensation was one of the most common complaints and was observed in more than 80% of patients with decreased MF, regardless of WF, and was improved by repeated stimulation with KDL. Organic abnormalities, such as wounds in the oral mucosa, were not observed in the study participants. In the absence of organic abnormalities, a burning sensation in the oral mucosa that lasts for more than 2 h a day for more than 3 months is defined as burning mouth syndrome (BMS) (48). BMS often occurs in older and middle-aged women and, owing to poorly understood etiologies, is difficult to treat (49). A burning sensation in the oral mucosa is associated with various accompanying symptoms, among which dry mouth has been reported to be the most common (50). The reduction in the burning sensation in the oral mucosa observed in this study might be due to the KDL-induced MF increase. Therefore, KDL may also be beneficial in the treatment of various oral problems that are associated with aging and may promote a healthy daily lifestyle in older individuals.

Even for healthy people, habitually gargling with KDL could have the effect of preventing each oral function, particularly the difficulty of swallowing and eating and the burning sensation in the oral mucosa, which are common problems in older individuals.

This study had several limitations. First, this was a single-center study conducted at a dental department in a Japanese university hospital, which included Japanese people who are familiar with kelp dashi; therefore, the generalizability of the findings to diverse domestic and international populations or settings may be restricted. Research subjects from countries other than Japan and/or subjects of non-Japanese ethnicity who are not familiar with KDL are needed. Second, only a few men were included in this study, and sex differences were not investigated. Sex differences in KDL effects need to be studied. Although this study included a small number of patients, the differences in the effects of KDL, including the improvement in the RT of the taste qualities and increased saliva secretion, were significant. The reason for the small number of patients was that the study setting involved a long-term, complex method cycle that combined different stimulation methods every 2 weeks. In the future, we plan to verify the KDL stimulation effects by increasing the number of participants using a simplified method and to report any new developments. Third, in this study that confirms the potential of KDL umami in older patients with xerostomia, subjects made fresh KDL regularly every 2 days at home using a common recipe that we provided them. Since KDL is rich in amino acids, which are nutrients that promote the growth of microorganisms, it spoils easily. We would like to develop the utility of the KDL effects into further therapeutic development research, in which it would be necessary to provide the subjects with homogenized KDL containing useful components. In the future studies, it is necessary to have comparative experiments between a group stimulated with KDL containing useful ingredients, a group stimulated with only glutamic solution, and a group stimulated with other taste solutions, such as glucose solution, to clarify the usefulness of KDL umami. Fourth, the relationship between age-related structural changes or damage, such as Sjögren's syndrome, in the minor salivary glands is unclear. Although the effects of aging on the minor salivary glands, such as structural degenerative changes, have been reported (51), salivary flow measurements in older people showed both decreased and unchanged function of the minor salivary glands (52–54). The relationship between sex, aging, diseases, and MF is yet to be conclusively clarified. In this study, we found that KDL repeated stimulation increased MF and WF both in the patients with Sjögren's syndrome and in the other patients. Various therapeutic options, including medications, transcutaneous electrical stimulation, and acupuncture, have been used to treat the dry mouth caused by Sjögren's syndrome. However, the available therapeutic options provide only limited benefits for these patients (55). KDL may promote the MF and the WF in patients with or without Sjögren's syndrome. Since the number of cases was small in this study, further research that increases the number of cases is needed. Furthermore, this study targeted the lower lip minor salivary glands, and differences in responses have been shown depending on the location of the minor salivary glands (18, 54). Therefore, it is also necessary to examine the different effects of KDL based on different locations of the minor salivary glands.

In conclusion, repeated stimulation with KDL improved taste decline and maintained normal taste sensation by increasing saliva production, as measured by MF and WF, in older adults through the umami-taste salivary reflex. Moreover, repeated stimulation with KDL alleviated subjective taste impairment, subjective eating difficulty, subjective swallowing difficulty, and burning sensations in the oral mucosa, all of which are daily eating and oral problems that plague older adults.

This study is the first to report these findings that emphasize the usefulness and contribution of KDL umami in improving taste decline, sustainably maintaining normal taste sensation, and promoting healthy eating habits in older adults.

## Data availability statement

The original contributions presented in the study are included in the article/supplementary material, further inquiries can be directed to the corresponding author.

## Ethics statement

This study was approved by the Ethics Committee of the Tohoku University Graduate School of Dentistry (Ethics No. 2016-3-027). The studies were conducted in accordance with the local legislation and institutional requirements. The participants provided their written informed consent to participate in this study.

## Author contributions

SS-K: Conceptualization, Data curation, Formal analysis, Funding acquisition, Investigation, Methodology, Project administration, Resources, Visualization, Writing – original draft, Writing – review & editing. SG: Methodology, Validation, Writing – review & editing. NS: Funding acquisition, Investigation, Formal analysis, Writing – review & editing. HU: Resources, Writing – review & editing. MK: Methodology, Supervision, Writing – review & editing.
